# Supplementary material for: Gamma activity accelerates during prefrontal development
Source: eLife. 2020 Nov 18;9:e56795. doi: 10.7554/eLife.56795 (PMC7673781; doi:10.7554/eLife.56795)
Supplement: Supplementary file 1. — Table summarizing the recordings for each experimental condition. [file elife-56795-supp1.docx]

**Supplementary file 1. Recording summary**

| **LFP analysis** | **Anesthetized**  **Head fixation acute**  **Acute recordings** | **Non-anesthetized**  **Head fixation acute**  **Acute recordings** | **Non-anesthetized**  **Head fixation implant**  **Acute recordings** | ***Total*** |
| --- | --- | --- | --- | --- |
| **P5-10** | 11 recordings / mice | 7 recordings / mice  (1 excluded from LFP baseline analysis due to artifacts) | - | *18 recordings* |
| **P11-15** | 15 recordings / mice | 3 recordings / mice | - | *18 recordings* |
| **P16-20** | 8 recordings / mice | - | - | *8 recordings* |
| **P21-25** | 22 recordings / mice | - | 9 recordings, 6 mice | *31 recordings* |
| **P26-30** | 6 recordings / mice | - | 4 recordings (same mice as for P21-25) | *10 recordings* |
| **P31-35** | 5 recordings / mice | - | - | *5 recordings* |
| **P36-40** | 13 recordings / mice | - | 12 recordings, 5 mice | *25 recordings* |
| ***Total*** | *80 recordings / mice* | *10 recordings / mice* | *25 recordings, 11 mice* | *115 recordings* |
|  |  |  |  |  |
| **SUA analysis** | **Anesthetized**  **Head fixation acute**  **Acute recordings** | **Non-anesthetized**  **Head fixation acute**  **Acute recordings** | **Non-anesthetized**  **Head fixation implant**  **Acute recordings** | ***Total*** |
| **P5-10** | 1 recordings / mice | 7 recordings / mice | - | *8 recordings* |
| **P11-15** | 10 recordings / mice | 3 recordings / mice | - | *13 recordings* |
| **P16-20** | 8 recordings / mice | - | - | *8 recordings* |
| **P21-25** | 13 recordings / mice | - | - | *13 recordings* |
| **P26-30** | 6 recordings / mice | - | - | *6 recordings* |
| **P31-35** | 5 recordings / mice | - | - | *5 recordings* |
| **P36-40** | 13 recordings / mice | - | - | *13 recordings* |
| ***Total*** | *56 recordings / mice* | *10 recordings / mice* | *-* | *66 recordings* |
|  |  |  |  |  |
| **SUA numbers** | **Both hemispheres**  **Regular spiking (RS)** | **Both hemispheres**  **Fast spiking (FS)** | **Stimulated hemisphere**  **Regular spiking (RS)** | **Stimulated hemisphere**  **Fast spiking (FS)** |
| **P5-10** | 315 | 4 | 191 | 2 |
| **P11-15** | 538 | 17 | 305 | 9 |
| **P16-20** | 489 | 59 | 273 | 37 |
| **P21-25** | 616 | 116 | 368 | 71 |
| **P26-30** | 428 | 70 | 238 | 39 |
| **P31-35** | 277 | 39 | 166 | 29 |
| **P36-40** | 509 | 77 | 283 | 39 |
| ***Total*** | *3172* | *382* | *1824* | *226* |
