# Supplementary material for: Gamma activity accelerates during prefrontal development
Source: eLife. 2020 Nov 18;9:e56795. doi: 10.7554/eLife.56795 (PMC7673781; doi:10.7554/eLife.56795)
Supplement: Supplementary file 2. — Table summarizing the statistical results. [file elife-56795-supp2.docx]

**Supplementary file 2. Detailed statistical results**

|  |  | **Description** | **Comment** | **Test** |  |  |  |
| --- | --- | --- | --- | --- | --- | --- | --- |
| **Figure 1** | d | Peak freq over age | per experiment - all | Mann-Kendall | n 114 | p 2.73E-08 | tau b 3.61E-01 |
|  | d | Peak ampl over age | per experiment - all | Mann-Kendall | n 114 | p 3.93E-22 | tau b 0.625 |
|  | d | Peak freq over age | per experiment - anesthetized | Mann-Kendall | n 80 | p 2.30E-07 | tau b 0.404 |
|  | d | Peak ampl over age | per experiment - anesthetized | Mann-Kendall | n 80 | p 1.77E-13 | tau b 0.571 |
|  | d | Peak freq over age | per experiment - non anesthetized | Mann-Kendall | n 34 | p 0.020 | tau b 0.288 |
|  | d | Peak ampl over age | per experiment - non anesthetized | Mann-Kendall | n 34 | p 6.13E-08 | tau b 0.671 |
|  | d | Linear regression model baseline LFP features to age | per recording  ANOVA  peak freq  peak ampl | n,df  114,112 | R^2^  0.55  df 1,111  1,111 | F  67.9  F 17.878  74.421 | p  5.48E-20  p 4.86E-05  5.05E-14 |
| **Figure 2** | a | PV over age | per experiment | Mann-Kendall | n 38 | p 1.29E-07 | tau b 0.623 |
|  | b | SOM over age | per experiment | Mann-Kendall | n 39 | p 0.990 | tau b -0.002 |
|  | h | FS proportion over age | per experiment | Mann-Kendall | n 66 | p 1.44E-07 | tau b 0.457 |
|  | i | half width over age - RS | per unit | Mann-Kendall | n 3172 | p 1.61E-28 | tau b -0.133 |
|  | i | half width over age - FS | per unit | Mann-Kendall | n 382 | p 5.17E-17 | tau b -0.295 |
|  | i | trough to peak over age - RS | per unit | Mann-Kendall | n 3172 | p 4.57E-05 | tau b -0.051 |
|  | i | trough to peak over age - FS | per unit | Mann-Kendall | n 382 | p 9.23E-11 | tau b -0.236 |
|  | i | amplitude over age - RS | per unit | Mann-Kendall | n 3172 | p 4.45E-22 | tau b -0.116 |
|  | i | amplitude over age - FS | per unit | Mann-Kendall | n 382 | p 3.82E-06 | tau b -0.162 |
|  | i | Linear regression model baseline unit features to age | per recording  ANOVA  ratio FS  half width RS  half width FS  trough peak RS  trough peak FS  ampl RS  ampl FS | n,df  52,44 | R^2^  0.551  df 1,44  1,44  1,44  1,44  1,44  1,44  1,44 | F  9.92  F 1.674  0.154  16.225  3.870  0.562  1.046  1.780 | p  2.31E-07  p 0.202  0.696  2.01E-04  0.055  0.457  0.311  0.188 |
| **Figure 3** | d | Peak freq over age 473nm | per experiment - all | Mann-Kendall | n 115 | p 7.69E-06 | tau b 0.2886 |
|  | d | Peak MI over age 473nm | per experiment - all | Mann-Kendall | n 115 | p 1.04E-09 | tau b 0.392 |
|  | d | Peak freq over age 473nm | per experiment - anesthetized | Mann-Kendall | n 80 | p 0.001 | tau b 0.251 |
|  | d | Peak MI over age 473nm | per experiment - anesthetized | Mann-Kendall | n 80 | p 6.06E-06 | tau b 0.351 |
|  | d | Peak freq over age 473nm | per experiment - non anesthetized | Mann-Kendall | n 35 | p 0.007 | tau b 0.333 |
|  | d | Peak MI over age 473nm | per experiment - non anesthetized | Mann-Kendall | n 35 | p 1.26E-04 | tau b 0.475 |
|  | d | Linear regression ramp features LFP model to age | per recording  ANOVA  peak freq  peak ampl | n,df  115,113 | R^2^  0.364  df 1,112  1,112 | F  33.6  F 13.962  43.897 | p  3.72E-12  p 2.95E-04  1.26E-09 |
| **Figure 3 - supplement 1** | c | Peak freq over age 594nm | per experiment - all | Mann-Kendall | n 111 | p 0.092 | tau b 0.110 |
|  | c | Peak MI over age 594nm | per experiment - all | Mann-Kendall | n 111 | p 0.741 | tau b 0.021 |
|  | c | Peak freq over age 594nm | per experiment - anesthetized | Mann-Kendall | n 76 | p 0.096 | tau b 0.133 |
|  | c | Peak MI over age 594nm | per experiment - anesthetized | Mann-Kendall | n 76 | p 0.811 | tau b 0.019 |
|  | c | Peak freq over age 594nm | per experiment - non anesthetized | Mann-Kendall | n 35 | p 0.520 | tau b 0.081 |
|  | c | Peak MI over age 594nm | per experiment - non anesthetized | Mann-Kendall | n 35 | p 0.988 | tau b -0.003 |
| **Figure 4** | d | RS rate change over age | per age group | Mann-Kendall | n 7 | p 0.071 | tau b 0.619 |
|  | d | FS rate change over age | per age group | Mann-Kendall | n 7 | p 0.880 | tau b 0.047 |
|  | e | RS MI hist over age | per unit | Mann-Kendall | n 1821 | p 1.52E-14 | tau b -0.122 |
|  | e | FS MI hist over age | per unit | Mann-Kendall | n 225 | p 0.906 | tau b 0.005 |
| **Figure 5** | b | Autocorrelation (stimulation) | Main factor RS / FS | 3x3 ANOVA | df 1 153093 | F 1.563 | p 0.211 |
|  | b | Autocorrelation (stimulation) | Main factor Age group | 3x3 ANOVA | df 6 153093 | F 25.440 | p 2.21E-30 |
|  | b | Autocorrelation (stimulation) | Main factor Frequency | 3x3 ANOVA | df 99 153093 | F 492.798 | p 0.000 |
|  | c | spike-triggered LFP (stimulation) | Main factor RS / FS | 3x3 ANOVA | df 1 815232 | F 277.206 | p 3.12E-62 |
|  | c | spike-triggered LFP (stimulation) | Main factor Age group | 3x3 ANOVA | df 6 815232 | F 847.458 | p 0.000 |
|  | c | spike-triggered LFP (stimulation) | Main factor Frequency | 3x3 ANOVA | df 400 815232 | F 614.590 | p 0.000 |
| **Figure 5 - supplement 1** | b | Autocorrelation (spontaneous) | Main factor RS / FS | 3x3 ANOVA | df 1 153093 | F 1.883 | p 0.169 |
|  | b | Autocorrelation (spontaneous) | Main factor Age group | 3x3 ANOVA | df 6 153093 | F 41.863 | p 2.53E-51 |
|  | b | Autocorrelation (spontaneous) | Main factor Frequency | 3x3 ANOVA | df 99 153093 | F 663.540 | p 0.000 |
|  | c | spike-triggered LFP (spontaneous) | Main factor RS / FS | 3x3 ANOVA | df 1 805201 | F 248.426 | p 5.83E-56 |
|  | c | spike-triggered LFP (spontaneous) | Main factor Age group | 3x3 ANOVA | df 6 805201 | F 1413.266 | p 0.000 |
|  | c | spike-triggered LFP (spontaneous) | Main factor Frequency | 3x3 ANOVA | df 400 805201 | F 747.008 | p 0.000 |
| **Figure 5 - supplement 2** | a | RS CV over age (spontaneous) | per age group | Mann-Kendall | n 7 | p 0.763 | tau b 0.142 |
|  | a | FS CV over age (spontaneous) | per age group | Mann-Kendall | n 7 | p 0.880 | tau b 0.047 |
|  | a | RS CV over age (stimulation) | per age group | Mann-Kendall | n 7 | p  0.880 | tau b 0.047 |
|  | a | FS CV over age (stimulation) | per age group | Mann-Kendall | n 7 | p 0.367 | tau b -0.333 |
|  | b | RS CV2 over age (spontaneous) | per age group | Mann-Kendall | n 7 | p 0.763 | tau b -0.142 |
|  | b | FS CV2 over age (spontaneous) | per age group | Mann-Kendall | n 7 | p 0.880 | tau b 0.047 |
|  | b | RS CV2 over age (stimulation) | per age group | Mann-Kendall | n 7 | p 0.367 | tau b -0.333 |
|  | b | FS CV2 over age (stimulation) | per age group | Mann-Kendall | n 7 | p 0.367 | tau b -0.333 |
|  | c | inter-spike interval (spontaneous) | Main factor RS / FS | 3x3 ANOVA | df 1 204893 | F 5.245 | p 0.022 |
|  | c | inter-spike interval (spontaneous) | Main factor Age group | 3x3 ANOVA | df 6 204893 | F 3519.858 | p 0.000 |
|  | c | inter-spike interval (spontaneous) | Main factor Frequency | 3x3 ANOVA | df 99 204893 | F 583.282 | p 0.000 |
|  | d | inter-spike interval (stimulation) | Main factor RS / FS | 3x3 ANOVA | df 1 204893 | F 146.645 | p 9.64E-34 |
|  | d | inter-spike interval (stimulation) | Main factor Age group | 3x3 ANOVA | df 6 204893 | F 2745.028 | p 0.000 |
|  | d | inter-spike interval (stimulation) | Main factor Frequency | 3x3 ANOVA | df 99 204893 | F 447.1624 | p 0.000 |
|  | e | pairwise phase consistency (spontaneous) | Main factor RS / FS | 3x3 ANOVA | df 1 20262 | F 1.149 | p 0.283 |
|  | e | pairwise phase consistency (spontaneous) | Main factor Age group | 3x3 ANOVA | df 6 20262 | F 6.835 | p 2.93E-07 |
|  | e | pairwise phase consistency (spontaneous) | Main factor Frequency | 3x3 ANOVA | df 9 20262 | F 164.038 | p 2.67E-301 |
|  | f | pairwise phase consistency (stimulation) | Main factor RS / FS | 3x3 ANOVA | df 1 20385 | F 3.619 | p 0.057 |
|  | f | pairwise phase consistency (stimulation) | Main factor Age group | 3x3 ANOVA | df 6 20385 | F 13.580 | p 1.90E-15 |
|  | f | pairwise phase consistency (stimulation) | Main factor Frequency | 3x3 ANOVA | df 9 20385 | F 124.882 | p 8.13E-230 |
| **Figure 6** | b | 10 to 1 ratio | Main factor RS / FS | 3x3 ANOVA | df 1 7698 | F 0.394 | p 0.530 |
|  | b | 10 to 1 ratio | Main factor Age group | 3x3 ANOVA | df 6 7698 | F 18.708 | p 1.00E-21 |
|  | b | 10 to 1 ratio | Main factor Stimulation frequency | 3x3 ANOVA | df 4 7698 | F 36.090 | p 6.29E-30 |
|  | d | RS rate change over age | per age group | Mann-Kendall | n 7 | p 0.035 | tau b 0.714 |
|  | d | FS rate change over age | per age group | Mann-Kendall | n 7 | p 0.071 | tau b 0.619 |
|  | f | RS rate change over age | per age group | Mann-Kendall | n 7 | p 0.006 | tau b -0.904 |
|  | f | FS rate change over age | per age group | Mann-Kendall | n 7 | p 0.071 | tau b -0.619 |
| **Other** |  |  |  |  |  |  |  |
|  |  | Linear regression peak freq baseline to ramp | per recording | n,df  114,112 | R^2^  0.16 | F  22.6 | p  6.00E-06 |
|  |  | Linear regression ampl baseline to ramp | per recording | n,df  114,112 | R^2^  0.206 | F  30.4 | p  2.30E-07 |
|  |  | Stepwise linear regression model unit features all to LFP peak freq ramp | per recording  ANOVA  half width FS  acorr peak RS  acorr peak FS | n,df  42,38 | R^2^  0.136  df  1,38  1,38  1,38 | F  3.15  F  5.721  0.214  0.012 | p  0.036  p  0.021  0.646  0.912 |
|  |  | Stepwise linear regression model unit features all to LFP ampl ramp | per recording  ANOVA  rate ramp RS  stLFP peak RS  pulse early RS  pulse late FS | n,df  48,43 | R^2^  0.716  df  1,43  1,43  1,43  1,43 | F  30.6  F  6.398  11.238  35.702  1.02E-05 | p  4.35E-12  p  0.015  1.67E-03  3.97E-07  0.997 |
